# Advanced recovery of ergosterol- and vitamin D_2_-rich fractions from mushrooms: process intensification and scale-up challenges

**DOI:** 10.3389/fnut.2026.1987834

**Published:** 2026-10-21

**Authors:** Kristian Pastor, Jelena Vladic, Nataša Nastić

**Affiliations:** 1 Faculty of Technology Novi Sad, University of Novi Sad, Novi Sad, Serbia; 2 LAQV-REQUIMTE, NOVA School of Science and Technology, NOVA University Lisbon, Caparica, Portugal; 3 Department of Chemical and Petroleum Engineering, Khalifa University of Science and Technology, Abu Dhabi, United Arab Emirates

**Keywords:** advanced extraction, deep eutectic solvents, edible mushrooms, ergosterol, process intensification, supercritical fluid extraction, UV photoconversion, vitamin D2

## Abstract

Vitamin D insufficiency remains widespread, intensifying interest in mushrooms as non-animal sources of vitamin D₂ and in their processing side streams as sustainable reservoirs of its precursor, ergosterol. This review evaluates the advanced recovery of ergosterol- and vitamin D₂-rich fractions from edible mushrooms, integrating feedstock preparation, extraction, UV photoconversion, process intensification, scale-up, environmental and economic performance, and patent activity. Although these approaches can improve recovery, selectivity, and processing efficiency, most evidence remains laboratory-based. Matrix properties, solvent use, energy delivery, light penetration, reactor design, and fractionation jointly determine product yield, stability, and scalability. The limited environmental and economic assessments currently available identify drying, electricity demand, solvent recovery, and purification as major feasibility constraints. Patent trends reveal a shift from simple mushroom irradiation toward integrated extraction–photoconversion–purification platforms, yet pilot-scale validation remains limited. Future research should prioritize complete process chains, specific solvent circularity, and integrated techno-economic and life-cycle assessment. Bridging these gaps is essential for translating mushroom-derived ergosterol and vitamin D₂ into scalable, nutritionally relevant, and sustainable food ingredients.

## 1 Introduction

Vitamin D is essential for calcium and phosphate homeostasis and skeletal mineralization, yet relatively few foods naturally provide nutritionally meaningful quantities. The European Food Safety Authority established an adequate intake of 15 μg day^−1^ for healthy individuals older than 1 year, assuming minimal cutaneous synthesis (1). Yet low vitamin D status remains widespread: a global analysis of 7.9 million participants found that 15.7% had serum 25-hydroxyvitamin D below 30 nmol L^−1^ and 47.9% below 50 nmol L^−1^ (2). Combined with the limited availability of naturally vitamin D-rich foods, this highlights the need for additional dietary sources.

Edible mushrooms are specific among non-animal foods because ultraviolet (UV) exposure can significantly increase their vitamin D₂ content. The US National Institutes of Health indicates that ½ cup of UV-exposed raw white mushrooms can provide approximately 9.2 μg (366 IU) vitamin D₂, equivalent to about 46% of the US Daily Value, whereas untreated mushrooms generally contain much lower amounts (3). This enrichment is based on the UV-induced conversion of ergosterol, the predominant sterol of fungal cell membranes, into previtamin D₂ and its subsequent thermal isomerization to vitamin D₂ (4, 5). Mushroom-derived vitamin D₂ is bioavailable, although its effect on total vitamin D status is not uniform because increases in serum 25-hydroxyvitamin D₂ may be accompanied by decreases in 25-hydroxyvitamin D₃ (6). Dietary modeling nevertheless suggests that regular consumption of UV-exposed button mushrooms could contribute substantially to recommended vitamin D intake (7). Additional non-animal sources may be also relevant in restrictive dietary patterns, where vitamin D adequacy requires particular attention (8).

The expanding mushroom industry also generates potentially valuable ergosterol-containing residual biomass. Global production within the broad category of mushrooms and truffles reached approximately 48.3 million tonnes in 2022, with China accounting for most reported production (9). Commercial grading, harvesting and processing can generate significant quantities of discarded biomass, including non-marketable caps, stipes, trimmings and irregular or undersized fruiting bodies for up to 30% of production mass (10, 11). These by-products retain ergosterol and other valuable constituents, making them promising feedstocks for bioactive recovery (5). Their composition and recovery potential depends on mushroom species, developmental stage, anatomical fraction, cultivation conditions, UV exposure, storage, drying and particle-size reduction (4, 5). Efficient recovery therefore requires consideration of feedstock characteristics and pretreatment alongside extraction conditions.

Conventional recovery of mushroom sterols generally involves organic-solvent extraction, frequently combined with saponification and liquid–liquid separation, and may require considerable solvent volumes, extended treatment time and multiple downstream operations (5). Consequently, ultrasound-assisted extraction (UAE), microwave-assisted extraction (MAE), pressurized liquid extraction (PLE), supercritical fluid extraction (SFE) and deep eutectic solvent or natural deep eutectic solvent (DES/NADES) systems have been investigated as alternatives. These technologies use different mechanisms to enhance mass transfer, shorten extraction time, reduce conventional solvent consumption or improve recovery and selectivity (12–17). MAE recovered ergosterol from white mushroom (*Agaricus bisporus*) processing by-products at a yield comparable to Soxhlet extraction, but within a substantially shorter time (14). Hydrophobic DESs have also been investigated for ergosterol extraction and purification, including solvent reuse over repeated extraction cycles (16). Comparative application of PLE and SFE showed that PLE favored overall sterol recovery, whereas SFE produced more highly enriched fractions (13). Integration of extraction and photoconversion represents a further development. Sequential SFE of shiitake mushroom (*Lentinula edodes*) followed by UV irradiation of the recovered ergosterol-rich extract produced vitamin D₂-enriched fractions (18). More recently, a NADES-based approach combined ergosterol extraction, UV-induced photoconversion and stabilization within an integrated process (19). However, integrated processing raises additional questions concerning light penetration, solvent effects on photochemical behavior, reactor geometry, separation of the enriched fraction and reuse of the extraction medium (18, 19).

Laboratory-scale feasibility does not ensure that an extraction method can be transferred directly to industrial operation. Scale-up can affect mass and energy transfer, acoustic or electromagnetic field distribution, solvent-to-feed requirements, and overall process efficiency (12, 15, 20). Extraction yield alone is therefore insufficient to establish industrial relevance. Productivity, selectivity, specific energy demand, solvent-to-feed ratio, solvent recovery, throughput, downstream separation and economic feasibility must also be considered. Vitamin D₂ production introduces additional scale-up challenges because photoconversion depends on UV wavelength and dose, exposure time, mushroom species, surface area, sample geometry and processing conditions, while excessive irradiation can generate lumisterol₂ and tachysterol₂ (4, 21). In ergosterol-rich SFE extracts from *L. edodes*, conversion depended on extract solubilization, irradiation wavelength, exposure distance and treatment duration (18), which should all be evaluated as interconnected operations.

Previous reviews have examined mushrooms as dietary sources of vitamin D, the nutritional effects of UV-irradiated mushrooms and their potential inclusion in dietary guidelines (4, 22). The occurrence, biosynthesis and nutraceutical relevance of ergosterol and vitamin D₂ have also been reviewed, including their presence in Himalayan mushrooms and recovery from mushroom residues (5, 23). However, limited attention has been given to comparing extraction approaches using process-relevant criteria or to examining extraction, photoconversion and downstream separation as an integrated production chain. Accordingly, this review evaluates conventional and advanced approaches for recovering ergosterol- and vitamin D₂-rich fractions from mushrooms and their processing side streams, with emphasis on advanced sample preparation and integrated strategies. The technologies are compared in terms of recovery, energy and solvent requirements, UV conversion and downstream processing. It further examines scale-up evidence, techno-economic and environmental feasibility, and the patent landscape to identify the principal barriers and research priorities for translating laboratory-scale methods into industrially viable production routes for mushroom-derived ergosterol- and vitamin D₂-rich ingredients.

## 2 Literature search and review methodology

This study was conducted as a narrative review focused on process-relevant evidence concerning the recovery and production of mushroom-derived ergosterol- and vitamin D₂-rich fractions. A literature search was conducted using Scopus, Web of Science, and Google Scholar to identify studies on the recovery of ergosterol- and vitamin D_2_-rich fractions from mushrooms. Its purpose was to integrate evidence combining food chemistry, extraction engineering, UV photochemistry, process intensification, scale-up, techno-economic assessment, life-cycle assessment, and patent activity. Literature published up to 1 September 2026 was considered. Search concepts combined mushroom terms (mushroom*, *Agaricus*, *Lentinula*, *Pleurotus*), target compounds (ergosterol, vitamin D_2_/ergocalciferol), and process terms (extraction, ultrasound, microwave, pressurized liquid, supercritical CO_2_, deep eutectic solvent, UV, photoconversion, scale-up, techno-economic, life-cycle assessment). Reference lists of relevant publications were also screened to identify additional studies.

Primary studies were eligible when they investigated edible mushrooms or mushroom-processing side streams and reported quantitative or process-relevant information on ergosterol/vitamin D₂ occurrence, pretreatment, extraction, photoconversion, fractionation, product stability, scale-up, process economics, or environmental performance. Reviews and methodological papers were used for scale-up, or methodological context. Studies without direct relevance to mushroom-derived ergosterol or vitamin D₂ processing were excluded from the process comparison.

The patent landscape was searched in Espacenet and Google Patents in August 2026 using combinations of “mushroom,” “ergosterol,” “vitamin D2,” “extraction,” “recovery,” “UV,” “irradiation,” and “photoconversion.” Records were retained when their claims or technical disclosure directly addressed mushroom-derived ergosterol or vitamin D₂ processing, enrichment, irradiation equipment, extraction, stabilization, or closely related downstream use. Legal status was checked at the search date where available, and withdrawn or pending applications were identified explicitly when relevant.

## 3 Ergosterol and vitamin D₂ in mushrooms

Ergosterol is the predominant sterol in fungal cell membranes and the principal endogenous precursor of vitamin D₂ in mushrooms (4, 21). Its concentration varies with mushroom species, strain, developmental stage, cultivation conditions, anatomical part and postharvest treatment (4, 5, 23). Untreated cultivated mushrooms generally contain limited vitamin D₂ because its formation largely depends on the exposure of ergosterol to UV radiation. UV irradiation induces photochemical opening of the ergosterol B-ring to form previtamin D₂, which subsequently undergoes thermal isomerization to vitamin D₂. UV-B is commonly considered effective, although UV-A and UV-C treatments have also been investigated (4, 24). However, continued irradiation can convert previtamin D₂ and vitamin D₂ into other photoproducts, including lumisterol₂ and tachysterol₂ (21, 25).

Vitamin D₂ concentration in mushrooms cannot be predicted from ergosterol content alone. In ten commercial mushroom types, mean ergosterol concentrations ranged from 26.3 to 84.9 mg 100g^−1^ fresh weight, whereas vitamin D₂ ranged from 0.06 to 28.1 μg 100g^−1^, with considerable variation among suppliers (26). Cultivated species may contain more ergosterol than wild species, while naturally exposed wild mushrooms frequently accumulate more vitamin D₂, demonstrating the importance of light exposure and production conditions (27, 28).

Ergosterol and vitamin D₂ are distributed unevenly within mushroom fruiting bodies, depending on species, anatomical fraction and developmental stage. In wild mushrooms, their concentrations differed among pilei, gills and stipes (27). The discarded lower stipe of *A. bisporus*, for example, contained 4.70 mg ergosterol g^−1^ dry weight, supporting the use of processing side streams as extraction feedstocks (13). Tissue orientation and geometry influence photon exposure: positioning *L. edodes* gills toward a UV-A source produced approximately 4.4-fold more vitamin D₂ than cap-surface exposure, while sliced *A. bisporus* accumulated more vitamin D₂ than whole mushrooms under UV-B irradiation (29, 30). More recent work indicates that particle size must be optimized rather than simply minimized because surface area, matrix porosity, optical penetration and light shielding interact to determine conversion efficiency (19).

Photoconversion depends on UV wavelength, irradiance, dose, spectral characteristics and matrix properties. UV-C at 254 nm increased vitamin D₂ in fresh *A. bisporus* and freeze-dried *Craterellus tubaeformis*, but also discolored white mushrooms (28). UV-A can also be effective under suitable irradiance, temperature and moisture conditions (29, 31). Under UV-B, increasing intensity can shorten treatment time when an equivalent dose is delivered, potentially improving throughput (32). Temperature and moisture also influence photoconversion. Under UV-A, vitamin D₂ formation varied among species and increased in *L. edodes* from 8.05 to 44.8 μg g^−1^ dry matter as temperature and moisture rose, although these responses may not apply universally (31).

The interacting biological, processing and analytical factors governing ergosterol photoconversion and the final measured vitamin D₂ concentration are summarized in Figure 1.

**Figure 1:**
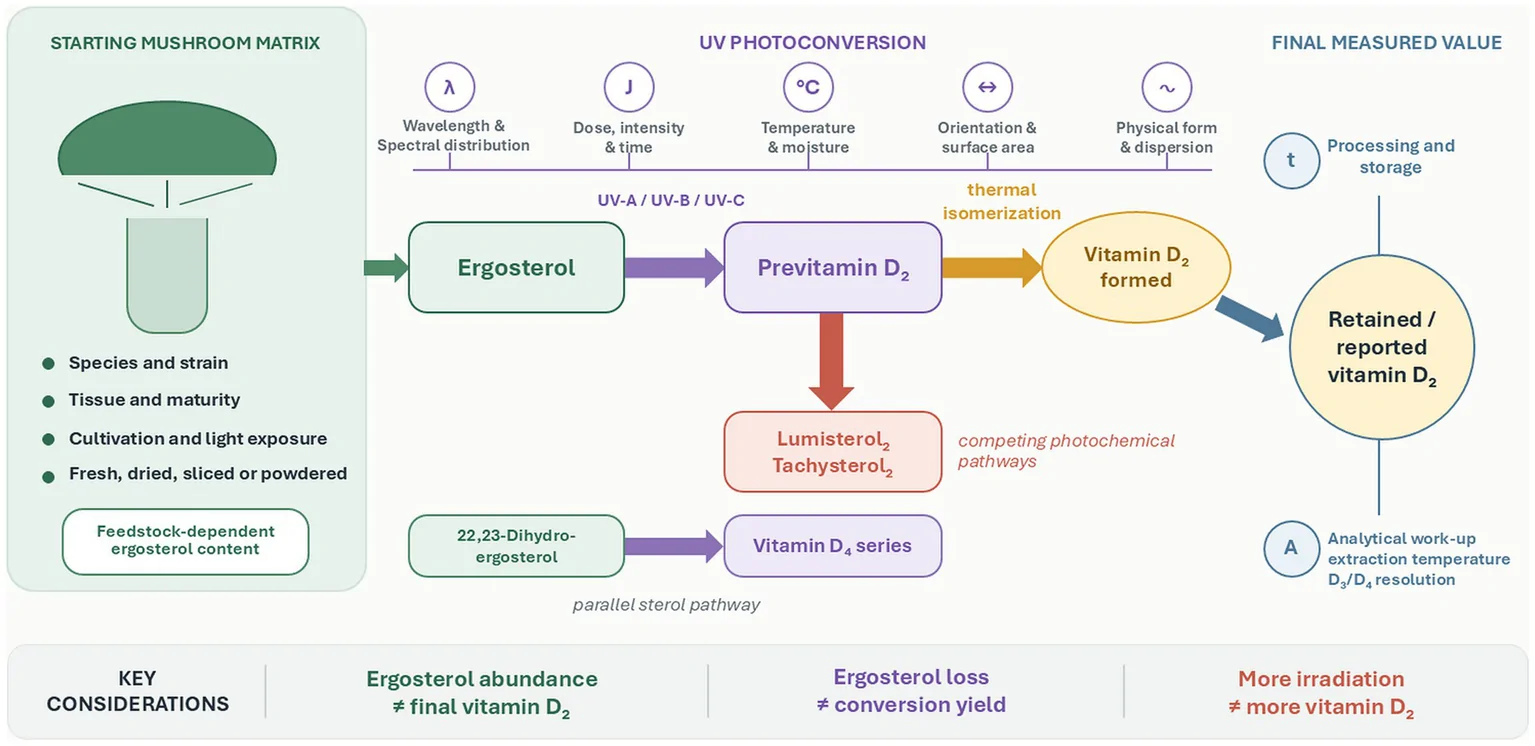
Biological, processing and analytical factors influencing ergosterol distribution and UV-induced vitamin D₂ formation in mushrooms.

Dehydrated mushrooms retain the capacity for ergosterol photoconversion, although the response depends on species, drying method and irradiation conditions. Freeze-dried mushrooms have generally produced more vitamin D₂ than hot-air-dried samples, possibly because their porous structure facilitated radiation penetration (30). Matrix dispersion can further enhance exposure. Continuously stirred ethanolic suspensions of dried *A. bisporus* and *Cordyceps militaris* powders accumulated approximately 15-fold more vitamin D₂ than directly irradiated powders. However, the prolonged 120-min treatment and 1:20 solid-to-liquid ratio limit the immediate scalability of this approach (33).

Longer irradiation time does not necessarily produce a proportional increase in vitamin D₂. In sliced *A. bisporus*, vitamin D₂ increased to 74.2 μg g^−1^ dry weight after 15 min of UV-B treatment, but declined to 34.4 μg g^−1^ after 30 min, and a similar response was observed in oyster mushroom (*Pleurotus ostreatus*) (30). Prolonged broad-spectrum irradiation of supercritical-fluid-extracted *L. edodes* fractions promoted photoproduct formation and eventually decreased both ergosterol and vitamin D₂ concentrations (18). UV-treated mushrooms may also contain vitamin D₄ and photoproducts derived from 22,23-dihydroergosterol (34, 35). These compounds are analytically relevant because vitamin D₄ may co-elute with vitamin D₃ used as an internal standard, making adequate chromatographic resolution essential.

Vitamin D₂ generated through irradiation is not completely stable during subsequent storage. After 18 months in darkness at 20 ± 2 °C, dried mushrooms retained from 48.3–68.4% of their initial vitamin D₂ concentrations, depending on species and processing conditions (30). Losses were also observed during four-days-refrigerated storage of fresh UV-B-treated *A. bisporus* Portobello (32), showing that concentrations determined immediately after irradiation may not represent those present at consumption or during subsequent ingredient processing.

Mushrooms therefore provide a controllable, non-animal source of vitamin D₂, and UV-treated *A. bisporus* powder has been considered safe for specified food and supplement applications by EFSA (36).

## 4 Mushroom matrices as extraction systems

Efficient recovery of ergosterol and vitamin D₂ depends not only on extraction technology, but also on the structural and physicochemical characteristics of the mushroom matrix. Mushroom species, strain, anatomical region, maturity, cultivation conditions, and postharvest process contribute to variability in sterol composition and extractability (5). Fungal cell walls contain a complex network dominated by chitin, β-glucans, proteins, and other polysaccharides that can restrict solvent penetration and intracellular mass transfer. Consequently, matrix disruption and pretreatment can influence the accessibility of membrane-associated ergosterol and should be considered integral components of the process (37). Cultivated species such as *A. bisporus*, *L. edodes*, *P. ostreatus*, and king oyster mushroom (*Pleurotus eryngii*) are notably relevant because they combine significant ergosterol contents with high commercial availability and generation of processing residues (Figure 2) (38–40).

**Figure 2:**
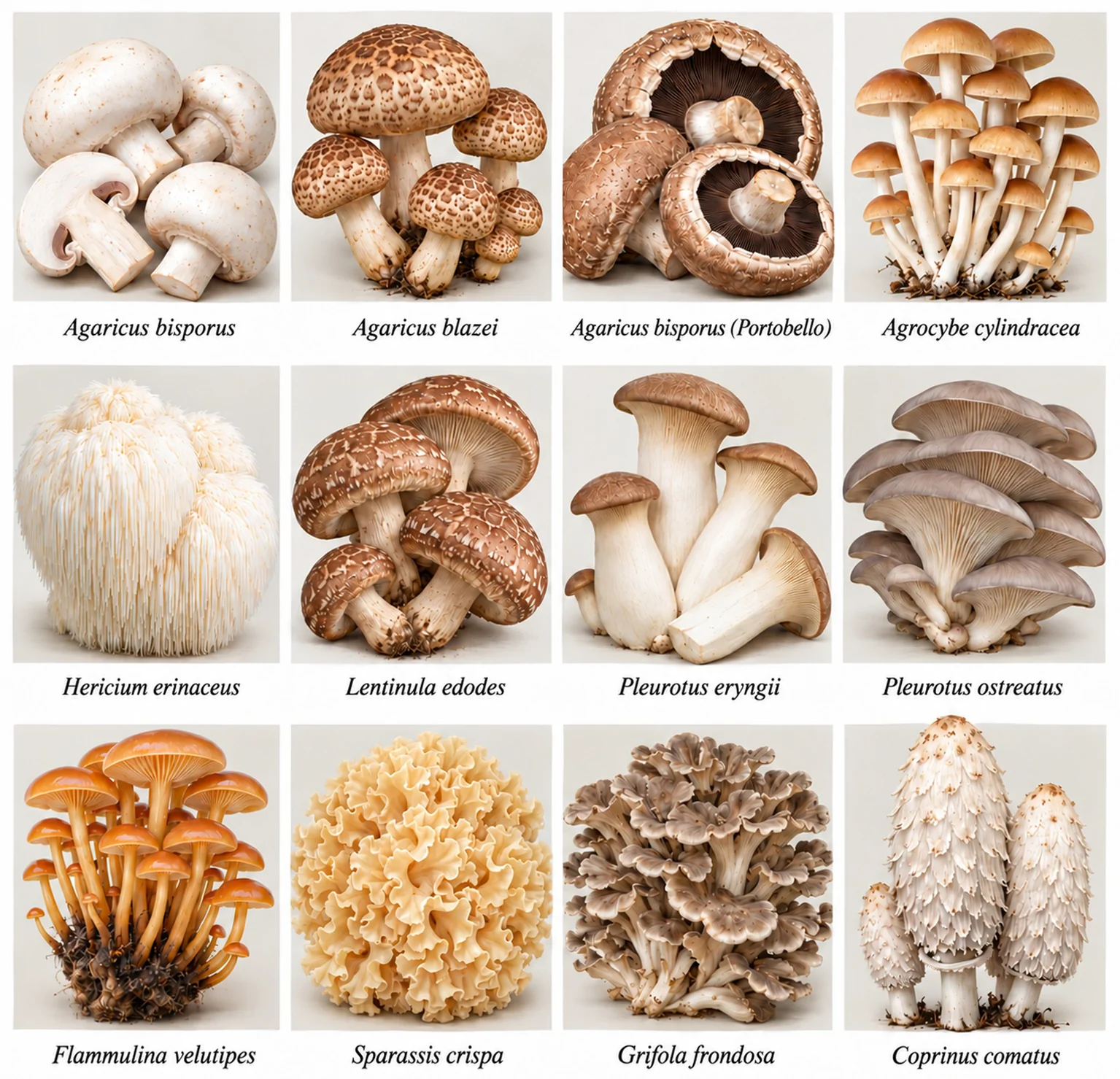
Selected commercially relevant mushroom species.

### 4.1 Drying and particle-size reduction

Fresh cultivated mushrooms typically contain approximately 90% moisture. For example, moisture contents of 90.8–91.3% were reported for *A. bisporus*, *A. bisporus* Portobello, and *P. ostreatus* (41). Drying is therefore commonly required before storage, milling, and extraction. However, drying conditions modify tissue structure, porosity, moisture distribution, and chemical stability, thereby influencing subsequent extraction performance. Hot-air drying at temperatures of 60–80 °C has been associated with significant losses of ergosterol and vitamin D₂ in mushrooms, with losses of approximately 30% (42). Therefore, freeze-drying is often preferred when minimizing thermal degradation of these compounds is important. The drying method also affects the composition of the resulting extraction feedstock. In *A. bisporus*, comparison of hot-air, freeze, and dehumidified drying demonstrated a significant effect of drying method on ergosterol retention, with dehumidified-dried mushroom powder containing 422.18 ± 5.80 mg ergosterol 100g^−1^ dry weight, compared with 324.54 ± 17.90 and 214.41 ± 3.20 mg 100g^−1^ following freeze-drying and hot-air drying, respectively (43). Recent research has focused on pretreatments that accelerate moisture removal while limiting thermal exposure (37, 44). Thermal and non-thermal approaches, including blanching, osmotic dehydration, ultrasound, pulsed electric fields, and pressure-based treatments, can modify cell-wall permeability and improve water transport, thereby reducing drying time. However, their benefits depend strongly on treatment intensity and mushroom structure (44). Drying is very important when vitamin D₂-rich fractions are targeted because ergosterol is photoconverted to vitamin D₂ and the resulting vitamin is sensitive to subsequent processing and storage. In UV-treated *A. bisporus*, air-dried mushrooms retain 100, 93, and 58% of vitamin D₂ after 3, 6, and 12 months, respectively, when vacuum-packed and stored in the dark at room temperature (4).

Following drying, milling further reduces mass-transfer limitations by increasing specific surface area and shortening the diffusion path between the intracellular sterol and extraction solvent. The importance of particle size was demonstrated recently for UAE of ergosterol from *Phlebopus portentosus*: an optimized particle size of 80 mesh, combined with methanol, a 1:30 g mL^−1^ solid-to-liquid ratio, 40 °C, and 30 min sonication, produced an ergosterol yield of 0.5283%, with the purified fraction exceeding 95% purity (45). In UV-B-treated *A. bisporus*, reducing the median particle diameter (Dv₅₀) from 231.0 to 106.3 and 7.1 μm increased specific surface area from 387 to 460 and 5,346 m^2^ kg^−1^, respectively. However, vitamin D₂ concentrations remained statistically unchanged at 7.6, 7.8, and 8.1 μg g^−1^, indicating that very fine jet milling preserved vitamin D₂, but did not further increase its concentration when milling was performed after irradiation (46). In contrast, when particle size is controlled before UV exposure, it can directly influence photoconversion efficiency. In *A. bisporus* powder, a median particle size of 130–140 μm combined with UV-B irradiation maximized vitamin D₂ yield at 2.59%, with the optimum attributed to a balance among optical penetration depth, light shielding, and the resulting porous cellular structure (19). Ultrafine grinding of UV-B-treated *Volvariella volvacea* increased cell-wall disruption and specific surface area and improved the solubility of both ergosterol and vitamin D₂, without disrupting the principal chemical structure of the mushroom powder (47). Ultrafine grinding combined with UV-C irradiation also enhanced ergosterol-to-vitamin D₂ bioconversion in *L. edodes* and *Auricularia auricula*, for which UV-C treatment increased vitamin D₂ from 1.38 to 20.11 μg g^−1^ and from 4.13 to 39.93 μg g^−1^, respectively (48). However, greater particle-size reduction may additionally increase energy consumption, fines formation and pressure drop during packed-bed extraction. Particle-size optimization should therefore be integrated with extraction and irradiation conditions rather than treated simply as a preliminary milling step.

### 4.2 Matrix-disruption pretreatments

Physical, thermal, enzymatic, and emerging non-thermal pretreatments can improve ergosterol recovery by disrupting the chitin-β-glucan-rich fungal cell wall and increasing accessibility of membrane-associated sterols. The most convincing evidence is provided by structural and enzymatic pretreatments that are physically separated from the subsequent extraction step.

More intensive structural disruption can produce substantial improvements in extraction kinetics. Liu et al. (49) applied steam explosion at 1.1 MPa for 7 min to golden needle mushroom (*Flammulina velutipes*) root waste before ultrasound-assisted saponification extraction. Pretreatment disrupted the compact fungal structure and increased porosity and surface roughness. The theoretical maximum ergosterol extraction concentration increased by 34.1%, while the apparent diffusion coefficient increased by 78.0%. Maximum ergosterol recovery was reached after approximately 20 min, compared with approximately 80 min for untreated material.

Saponification is frequently incorporated before or after solvent extraction to remove interfering lipids and release sterols from complex matrices. A typical procedure involves alkaline treatment followed by liquid–liquid extraction of the unsaponifiable fraction into a non-polar solvent (5). Although this can improve sterol isolation and analytical selectivity, it adds heating, reagents, solvent handling, phase separation, and wastewater-generating operations. Importantly, Heleno et al. (14) reported that saponification could be omitted without a significant reduction in ergosterol recovery under their optimized conditions, suggesting that elimination of this step may simplify processing when sufficiently selective extraction is achieved.

Recent SFE experiments with *L. edodes* and *P. ostreatus* evaluated ultrasound, microwave, and enzymatic pretreatments before high-pressure extraction. Chitinease pretreatment was more effective, increasing the ergosterol concentration of the *L. edodes* extract to approximately 338 mg g^−1^ extract (~20% more) under optimized SFE conditions (50). This finding demonstrates that selective disruption of the chitin-containing fungal matrix can improve accessibility of membrane-associated ergosterol and suggests opportunities for coupling biological pretreatment with intensified extraction. The same study provides a useful comparison with physical pretreatments. Microwave and ultrasound treatment affected subsequent SFE performance, but their effects were species- and treatment-dependent and were less consistently beneficial than chitinase treatment. This observation is important for process design: greater physical energy input does not necessarily produce greater sterol recovery, because extraction depends on the interaction between the pretreatment-induced structural changes and the subsequent solvent transport mechanism (50).

## 5 Extraction techniques for ergosterol and vitamin D₂ recovery

### 5.1 Conventional recovery as the technological benchmark

Conventional recovery of ergosterol and vitamin D₂ from mushrooms relies predominantly on solid–liquid extraction using organic solvents, with Soxhlet extraction, maceration, and heat-assisted extraction serving as established approaches (Table 1) (5). Owing to the lipophilic nature of sterols, solvents such as ethanol, methanol, hexane, chloroform, or their mixtures have been used, with extraction efficiency strongly dependent on solvent polarity, temperature, extraction time, and solid-to-solvent ratio (5). These methods remain useful reference points because they provide comparatively simple and well-established recovery procedures against which intensified technologies can be assessed.

**Table 1:** Conventional extraction techniques for ergosterol recovery from mushrooms.

Mushroom/material	Pretreatment	Extraction technique	Solvent	Temperature (°C)	Time (min)	Ergosterol recovery/content (mg 100 g^−1^ dw)	Ergosterol concentration in extract/purity (mg g^−1^)	Ref.
*A. bisporus*	Freeze-dried, milled	Soxhlet	Ethanol	Reflux	240 (12 cycles)	676	56.3	(14)
	Freeze-dried, milled	Soxhlet	*n*-Hexane	Reflux	240 (12 cycles)	186.1	108.8	(14)
	Freeze-dried, milled	Soxhlet	Limonene	Reflux	240 (12 cycles)	261	3.39	(14)
	Freeze-dried, milled	Soxhlet	*n*-Hexane	Reflux	240 (25 cycles)	365	238.32	(39)
	Freeze-dried, milled	Soxhlet	Ethanol	Reflux	240 (28 cycles)	544	56.83	(39)
*A. bisporus* (stalk by-products)	Fresh	Mechanical agitation	70 and 96% ethanol	25	30	70–200	NR	(53)
*A. blazei* (discarded fruiting bodies)	Dried, milled	Soxhlet	Ethanol	Reflux	240 (5 cycles)	0.058	4.80	(69)
	Oven dried, ~40 mesh	HAE	Ethanol	81.6	150	18.84	1.21	(52)
*Agrocybe cylindracea* (bio-residue)	Oven-dried, ~40 mesh	HAE	Ethanol	90	150	329.79	38.76	(70)
*Hericium erinaceus* (bio-residue)	Freeze-dried, ~100–200 mesh	SLE	Ethanol	25	3 days	252	26.77	(71)
*P. ostreatus*	Oven dried, ~40 mesh	HAE	Ethanol	54.3	150	244.25	33.32	(72)
*P. ostreatus* (bio-residue)	Oven-dried, ~40 mesh	HAE	Ethanol	90	50.4	290.38	27.58	(70)
*P. eryngii*	Oven dried, ~40 mesh	HAE	Ethanol	61.8	150	360.58	43.63	(72)

Soxhlet extraction represents a relevant benchmark for ergosterol recovery. In *A. bisporus*, Heleno et al. (14) evaluated ethanol, n-hexane, and limonene using approximately 4 h of Soxhlet extraction. Ethanol produced high ergosterol recovery, whereas n-hexane generated extracts with greater ergosterol purity because of its affinity for lipophilic constituents (14). However, conventional Soxhlet processing requires prolonged heating and relatively high solvent volumes. More recent work confirms this characteristic of Soxhlet extraction. Almeida et al. (39) reported that Soxhlet extraction of *A. bisporus* with n-hexane for 4 h (25 cycles) provided around 4.2-fold greater ergosterol purity than ethanol (238 vs. 56.8 mg g^−1^ extract), while ethanol gave greater recovery per mass of mushroom (28 h).

The principal limitations of conventional extraction are therefore not necessarily inadequate recovery, but long extraction times, high solvent-to-solid ratios, thermal exposure, solvent evaporation, and multiple downstream operations (5). These factors become increasingly important during scale-up because solvent consumption and recovery influence equipment size, energy requirements, operating cost, environmental performance, and process safety. For food applications, solvent selection is additionally constrained by food compatibility and the need to minimize residual solvents.

### 5.2 Advanced extraction technologies

Advanced extraction technologies aim to overcome the long extraction times, high solvent consumption, and limited selectivity associated with conventional recovery. For mushroom sterols, the most investigated approaches include UAE, MAE, PLE, SFE, and DES/NADES-based extraction (Table 2).

**Table 2:** Advanced extraction techniques for ergosterol recovery from mushrooms.

Mushroom/material	Pretreatment	Extraction technique	Solvent	Temperature (°C)	Time (min)	Pressure/power	Ergosterol recovery/content (mg 100 g^−1^ dw)	Ergosterol concentration in extract/purity (mg g^−1^)	Ref.
*A. bisporus*	Freeze-dried, 20 mesh	UAE	Ethanol	NR	5	375 W	671.53	36.72	(14)
	Freeze-dried, 20 mesh	UAE	n-Hexane	NR	5	375 W	152.2	113.9	(14)
	Freeze-dried, 20 mesh	UAE	Limonene	NR	5	375 W	372.0	12	(14)
	Freeze dried, milled	HDES	20 mL of DL-menthol:pyruvic acid (1:2)	NR	120 (stirring) + 45 (sonication)	NR	~699.5	NR	(16)
	Freeze-dried, milled	SFE	CO₂ + 8% ethanol	56	NR	24.4 MPa	623	311.80	(39)
	Freeze-dried, milled	SFE	CO₂ + 5% ethanol	50	NR	10 MPa	597	547.27	(39)
	Freeze-dried, 20 mesh	UAE	Ethanol	40	30	40 kHz, 240 W	680	NR	(41)
*A. bisporus* Portobello	Freeze-dried, 20 mesh	UAE	Ethanol	40	30	40 kHz, 240 W	730	NR	(41)
*A. bisporus* (bio-residue)	Freeze-dried, 20 mesh	MAE	Ethanol	131.6	20.4	400 W	555.3	13.2	(55)
*A. bisporus* (stalk by-products)	Fresh	UAE	70 and 96% ethanol	25	30	182 and 321 W L^−1^	157–277	NR	(53)
*A. blazei* (discarded fruiting bodies)	Oven dried, ~40 mesh	UAE	Ethanol	30–35	30	400 ± 18.7 W	21.49	1.77	(52)
	Oven dried, ~40 mesh	MAE	Ethanol	134.6	25	400 W	25.44	1.01	(52)
*F. velutipes* (root)	Steam explosion (1.1 MPa, 7 min); oven-dried	UASE	95% ethanol + KOH (2 mol L^−1^)	25	30	500 W; 40 kHz	169	NR	(49)
*G. lucidum*	Freeze-dried; milled	SFE	CO₂	50	~84	35 MPa	NR	5.23	(58)
*H. erinaceus* (bio-residue)	Freeze-dried, ~100–200 mesh	SFE	CO₂	70	40	35 MPa	111	61.15	(71)
	Freeze-dried, ~100–200 mesh	SFE	CO₂ + ethanol (0.99 mL min^−1^)	46.38	60	10 MPa	393	37.14	(73)
*L. edodes*	Freeze-dried, 80 mesh	UASE	Methanol + KOH (0.04 g mL^−1^)	70	70	400 W	810	861.8	(74)
	Dried, 60–230 mesh	SFE	CO₂	69.8	180	69 MPa	353.53	280.57	(50)
	Dried, 60–230 mesh; Chitinase pretreatment	SFE	CO₂	69.8	180	69 MPa	919.34	337.53	(50)
*L. edodes* (stems)	Freeze-dried, 80 mesh	NADES-UAE	Pro-LeA	50	80	175 W	176	NR	(60)
*P. ostreatus*	Dried, 60–230 mesh	SFE	CO₂	69.8	180	69 MPa	215.69	158.21	(50)
	Freeze-dried, 20 mesh	UAE	Ethanol	40	30	40 kHz, 240 W	490	NR	(41)

NR, not reported; Pro-LeA, L-Proline and Levulinic acid; UASE, Ultrasound-assisted saponification extraction.

#### 5.2.1 Ultrasound-assisted extraction

UAE enhances solid–liquid extraction through acoustic cavitation. The formation, growth, and collapse of cavitation bubbles generate localized shear forces, microjets, and turbulence that can disrupt plant tissues, facilitate solvent penetration, decrease external mass-transfer resistance, and accelerate the release of intracellular compounds (51). The potential of UAE for recovering mushroom sterols has been demonstrated in *A. bisporus* by Heleno et al. (14). The authors optimized UAE of mycosterols from *A. bisporus* using response surface methodology and compared the optimized procedure with conventional Soxhlet extraction. Their results demonstrated that UAE could effectively recover ergosterol while substantially reducing the severity and duration of the conventional extraction process. Subsequent comparative work with almond mushroom (*Agaricus blazei*) similarly showed that UAE improved ergosterol extraction relative to conventional heat-assisted approaches (52).

More detailed mechanistic evidence was provided by Umaña et al. (53) using *A. bisporus* stalks, an industrial mushroom-processing by-product. At 25 °C, ultrasound treatments with acoustic power densities of 182 and 321 W L^−1^ accelerated the extraction of ergosterol, phenolic compounds, and antioxidant components compared with mechanical agitation. Depending on ethanol concentration and acoustic power density, ergosterol extraction under ultrasound was increased by as much as approximately twofold. Solvent composition was also decisive: 96% ethanol produced approximately twice the ergosterol yield obtained with 70% ethanol. Thus, cavitation-induced matrix disruption cannot be considered independently of solvent–solute affinity. Efficient UAE requires simultaneous optimization of both physical intensification and solvent composition. Importantly, the residue remaining after extraction retained a high β-glucan content, suggesting that UAE can be incorporated into sequential biorefinery schemes in which ergosterol-rich extracts and polysaccharide-rich solid fractions are recovered from the same mushroom by-product.

The sensitivity of UAE to operating conditions is further illustrated by analytical optimization studies. For freeze-dried *A. bisporus*, *A. bisporus* Portobello, and *P. ostreatus*, optimized conditions of 30 min, 40 °C, 40 kHz, 240 W, and a solid concentration of 20 mg mL^−1^ were reported, with ethanol identified as the most suitable solvent for simultaneous extraction of ergosterol and vitamin D₂ (41). From a processing perspective, UAE has several advantages, including relatively mild bulk temperatures, short extraction times, compatibility with food-grade ethanol, and the possibility of integrating ultrasonic devices with existing solid–liquid extraction operations.

#### 5.2.2 Microwave-assisted extraction

MAE enhances extraction through rapid interaction of microwave radiation with polar and ionic constituents of the solvent-matrix system. Dipolar rotation and ionic conduction generate volumetric heating, allowing rapid temperature increases within the extraction medium and hydrated biological tissues (54). The resulting thermal and pressure effects can increase solvent diffusivity, weaken matrix structures, and accelerate the transfer of ergosterol into the surrounding solvent. For *A. bisporus* processing by-products, Heleno et al. (55) optimized MAE as a function of time (3–20 min), temperature (60–210 °C), and solid-to-liquid ratio (1–20 g L^−1^). Optimal conditions of approximately 19.4 min, 132.8 °C, and 1.6 g L^−1^ produced 556.1 mg ergosterol 100g^−1^ dry mushroom by-product, achieving recovery comparable to conventional extraction within a significantly shorter extraction period (55). Taofiq et al. (52) provided a direct comparison of MAE with UAE and HAE for recovering ergosterol from *A. blazei* bioresidues. MAE substantially reduced the extraction time relative to conventional heating (25 min vs. 150 min), although UAE was identified as the most appropriate technique for obtaining extracts with the highest ergosterol content. However, the relatively high temperatures used in optimized laboratory MAE procedures require consideration when vitamin D₂ rather than ergosterol is the final target, because process optimization must account for compound stability as well as extraction yield. Continuous-flow microwave systems may overcome some limitations of batch operation, but mushroom-specific evidence at pilot or industrial scale remains limited.

#### 5.2.3 Pressurized liquid extraction

PLE uses liquid solvents at elevated temperature and pressure to improve solubility, diffusivity, and matrix penetration while maintaining the solvent in the liquid state (56). Unlike UAE and MAE, relatively few studies have specifically examined PLE for mushroom sterol recovery. Gil-Ramírez et al. (13) demonstrated that ethanol-based PLE could produce sterol-enriched fractions from *A. bisporus* fruiting bodies and by-products. At 10.7 MPa, five 5-min extraction cycles generated extracts containing approximately 5% total sterols at 50 °C and 2.9% at 100 °C, showing that increasing extraction temperature does not necessarily improve sterol enrichment (13).

PLE enables short extraction cycles, reduced solvent consumption relative to prolonged conventional extraction, compatibility with food-grade ethanol, and precise control of temperature, pressure, solvent composition, extraction cycles, and solvent-to-feed ratio, facilitating optimization of extraction efficiency and selectivity. However, these benefits should be considered alongside the requirements for pressure-rated equipment, energy for heating and pressurization, solvent recovery, and safe depressurization.

#### 5.2.4 Supercritical fluid extraction

SFE, particularly using supercritical CO₂ (scCO₂), is effective for recovering relatively non-polar mushroom constituents such as sterols (18). Above its critical point, CO₂ combines gas-like diffusivity with liquid-like solvating properties, while its solvent strength and the composition of the resulting lipophilic fractions can be influenced by extraction conditions (57). In addition, CO₂ is non-flammable and readily removed by depressurization, while polar cosolvents such as ethanol can enhance the recovery of compounds with limited solubility in pure scCO₂.

Early work demonstrated the feasibility of producing sterol-enriched *A. bisporus* fractions using compressed-fluid technologies (13). More recently, Milovanovic et al. (58) demonstrated that the composition and origin of reishi (*Ganoderma lucidum*) can influence scCO₂ extraction outcomes. Cultivated samples contained higher concentrations of ergosterol (5.23 mg g^−1^ extract) in comparison to wild *G. lucidum* (2.27 mg g^−1^ extract). Almeida et al. (39) further optimized SFE of ergosterol from *A. bisporus* using response surface methodology and evaluated pressure, temperature, ethanol cosolvent fraction, and solvent-to-mushroom ratio against three distinct responses: total extraction yield, ergosterol recovery, and ergosterol purity. Authors reported that increasing ethanol enhanced overall extraction, but could reduce ergosterol purity because additional matrix constituents were co-extracted. The highest reported ergosterol purity, approximately 547 mg ergosterol g^−1^ extract, was obtained at 10 MPa, 50 °C, and 5% (v/v) cosolvent, whereas the highest ergosterol recovery, approximately 623 mg 100g^−1^ dry mushroom, occurred at about 24.4 MPa, 56 °C, and 8% (v/v) cosolvent. Thus, the operating point that maximizes product purity differs from that maximizing recovery. Further evidence was provided by Faustino et al. (50), who optimized scCO₂ extraction of ergosterol from *L. edodes* and subsequently applied the process to *P. ostreatus*. Optimized SFE at 69 MPa and 69.8 °C without a cosolvent produced ergosterol concentrations of 280.57 mg g^−1^ extract for *L. edodes* and 95.87 mg g^−1^ extract for *P. ostreatus*, representing increases of 107 and 65%, respectively, compared with Soxhlet extraction. The study also investigated ultrasound, microwave, and enzymatic pretreatments prior to SFE. Among these, chitinase treatment was notably effective, increasing the ergosterol concentration in *L. edodes* extracts to 337.53 mg g^−1^ extract. These results indicate that coupling biomass pretreatment with scCO₂ extraction may provide an effective strategy for producing concentrated sterol fractions (50).

SFE enables selective recovery of lipophilic compounds with minimal solvent residues, moderate operating temperatures, tunable selectivity and the possibility of fractionation during depressurization. For ergosterol recovery, high extract yield is not necessarily desirable because co-extraction of non-target compounds can reduce purity and increase downstream purification requirements. However, SFE requires high-pressure equipment, CO₂ compression, and potentially long extraction times, while cosolvent use introduces additional recovery requirements. Consequently, SFE is suitable for producing high-purity, food- or nutraceutical-grade sterol fractions rather than maximizing total extract yield.

#### 5.2.5 DES and NADES-based extraction

DES and NADES have emerged as alternative extraction media whose physicochemical properties can be adjusted through the selection and ratio of their constituent hydrogen-bond donors and acceptors (59). Hydrophobic systems are particularly relevant to ergosterol because of its lipophilic character and limited compatibility with highly aqueous extraction media.

Khare et al. (16) provided an early mushroom-specific application by screening 39 hydrophobic DES formulations for ergosterol extraction from *A. bisporus* and optimizing a menthol:pyruvic acid system using response surface methodology. The recovered ergosterol was purified and converted to vitamin D₂ by UV-B irradiation. The HDES was reused over six consecutive extraction cycles, although extraction efficiency declined with repeated use. The study therefore demonstrated the potential of hydrophobic DES as part of a broader strategy combining ergosterol recovery with subsequent vitamin D₂ production.

More recent studies have combined DES/NADES extraction with physical intensification. Liu et al. developed an ultrasound-assisted NADES process for ergosterol recovery from *L. edodes* stems. Among the systems investigated, proline:levulinic acid showed the highest extraction efficiency, while NADES extraction achieved higher efficiency than the conventional organic solvents evaluated in the study. Structural observations by SEM indicated greater disruption of the mushroom matrix following NADES treatment, consistent with improved release of ergosterol (60). The NADES could be recycled for seven extraction cycles without a significant decrease in extraction efficiency.

A further level of process integration was demonstrated by Yu et al. (19), who developed a menthol-based NADES system in which the same medium was used for ergosterol extraction and subsequent UV-induced conversion to vitamin D₂. The optimized system achieved an ergosterol extraction efficiency of 85.41% and a vitamin D₂ yield of 4.08%, while also improving the storage stability of both compounds relative to ethanol (19). Importantly, the NADES-containing supernatant obtained after UAE was used directly as the reaction medium for photoconversion, avoiding isolation of ergosterol between these two operations. This approach therefore extends process intensification beyond extraction itself by integrating ergosterol recovery and photoconversion within a single processing strategy.

### 5.3 Process intensification through hybrid and integrated approaches

Most studies on mushroom sterol recovery have optimized extraction, photoconversion, and downstream processing as separate operations. Process intensification provides a different approach by combining complementary mechanisms or integrating unit operations to enhance mass and photon transfer while reducing processing time, solvent use, and intermediate steps.

Physical treatments can improve photoconversion by increasing the accessibility of membrane-associated ergosterol to UV radiation. Chen et al. (61) combined microwave and ultrasound pretreatment with UV irradiation of 4D-printed formulations containing *L. edodes* residues. Vitamin D₂ concentrations in the printed products were up to 4.6-fold higher than in the raw material, while physical-field pretreatment produced 2.2–3.8-fold higher vitamin D₂ levels than corresponding samples without pretreatment. A more direct intensification strategy is to couple ergosterol extraction with UV-mediated vitamin D₂ formation. Conventional processes generally follow either UV irradiation before extraction or extraction followed by irradiation. Direct irradiation minimizes solvent handling, but can be limited by poor UV penetration through the mushroom matrix. Irradiation after extraction increases precursor accessibility, but introduces additional solvent-dependent photochemistry and intermediate handling. Sun et al. (25) compared *A. bisporus* powder, ethanol suspension, and extract during UV-C treatment. The extract provided the most effective photoconversion, with approximately 0.95–1.03 mg vitamin D₂ g^−1^ dry weight obtained at 0.31 mW cm^2-1^ for 10 min. The study also showed that *A. bisporus* contained approximately 3–8 mg ergosterol g^−1^ dry weight, providing a substantial precursor source. However, prolonged or more intense irradiation increased formation of the photoisomers lumisterol and tachysterol. Morales et al. (18) demonstrated a sequential extraction-photoconversion strategy for producing vitamin D₂-enriched extracts from *L. edodes*. SFE concentrated fungal sterols, with pressure affecting extraction yield and ergosterol concentration more strongly than temperature. The extracts were dissolved in methanol or ethanol and UV-irradiated, with 254 nm converting most ergosterol into vitamin D₂ more effectively than 365 nm. Although full-spectrum UV produced the fastest conversion, it also generated vitamin D₄ and lumisterol₂. Thus, the process represents a hybrid configuration that improves precursor accessibility, but retains separate extraction, solvent-redissolution, and irradiation steps. Process intensification can also be achieved by modifying the physical configuration of the mushroom matrix. Yu et al. (19) demonstrated that controlling *A. bisporus* powder particle size to approximately 130–140 μm produced a vitamin D₂ yield of 2.59% under UV-B treatment. The optimum reflected a balance among increased accessible surface area, optical penetration, light shielding, and matrix porosity. Intensification of photon delivery itself is another emerging strategy. Jung et al. (24) investigated intense pulsed light (IPL) as an alternative to continuous UV treatment of *L. edodes* powder. Vitamin D₂ increased from 4.18 to 100.82 μg g^−1^ under the maximum reported condition (1800 V, 30 min), representing approximately a 24-fold increase. IPL also produced greater vitamin D₂ enrichment than continuous UV treatment (24). Yu et al. (19) developed a menthol-based NADES system for *A. bisporus* that simultaneously supported ergosterol extraction, UV-B photoconversion, and stabilization. The optimized system achieved 85.41% ergosterol extraction efficiency and a 4.08% vitamin D₂ yield. Relative to ethanol, the NADES increased storage stability of ergosterol and vitamin D₂ by 24.79 and 20.53%, respectively, effects attributed partly to enhanced cell permeability and hydrogen-bonding interactions within the solvent system (19).

The principal distinction between a hybrid process and an intensified process is therefore functional rather than technological. Adding pretreatments or another operation is advantageous only when the resulting gain in mass transfer, photon accessibility, selectivity, or throughput exceeds the additional energy and equipment burden. Greater intensification is achieved when one processing step performs several functions, for example, matrix disruption and extraction, or extraction, photoconversion, and stabilization, thereby eliminating intermediate operations.

For mushroom-derived ergosterol and vitamin D₂, increasing integration can be conceptualized as conventional, hybrid and integrated, as shown in Figure 3. The emerging evidence therefore shifts the optimization target from maximum ergosterol extraction or vitamin D₂ concentration toward overall process efficiency.

**Figure 3:**
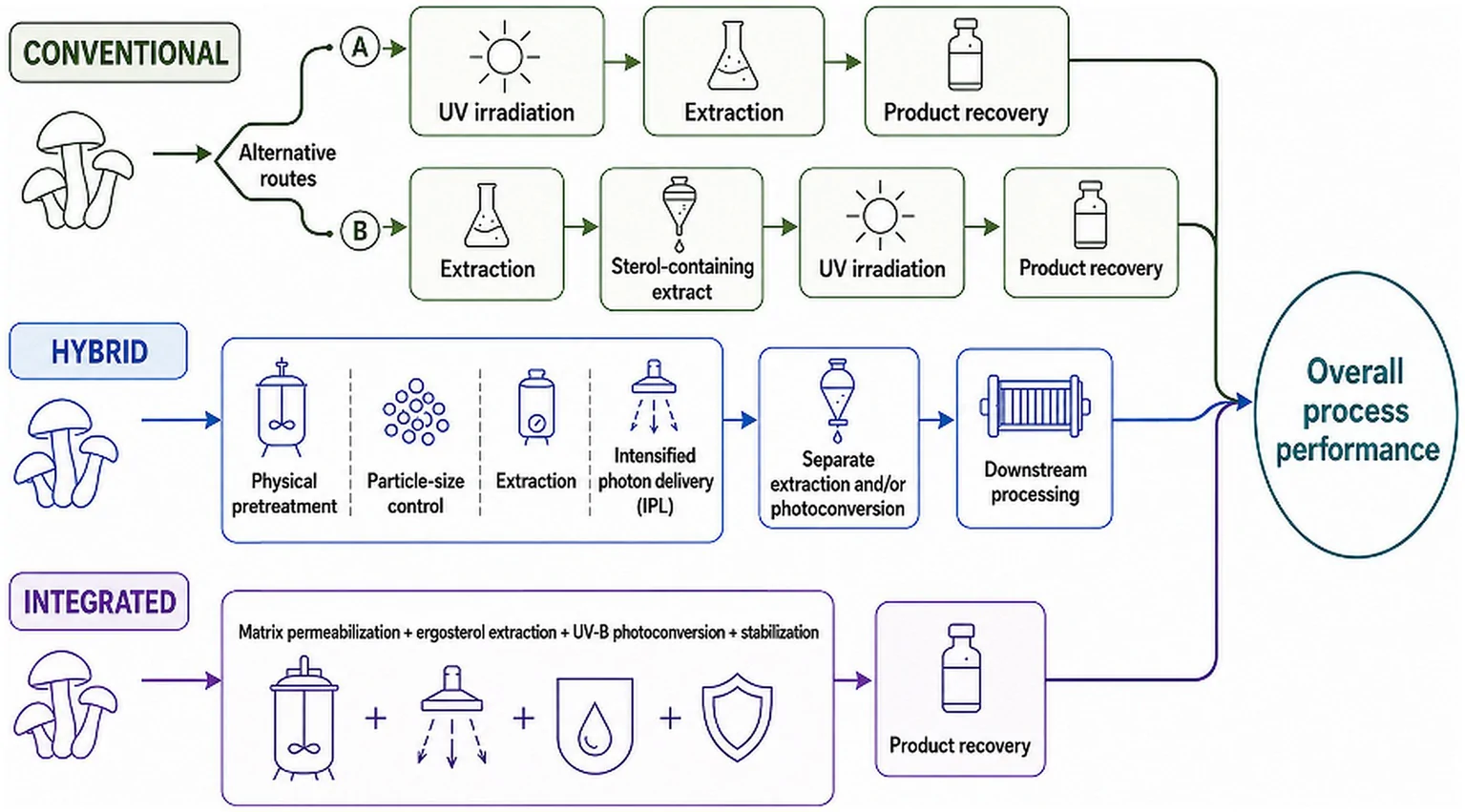
Representative conventional, hybrid and integrated routes for mushroom ergosterol recovery and vitamin D₂ production. The pathways are illustrative rather than exhaustive and may vary in their operations.

## 6 The scale-up gap

The advanced extraction and process-intensification strategies discussed above demonstrate that high ergosterol recovery and selectivity can be achieved under carefully optimized conditions, while controlled UV irradiation can efficiently promote ergosterol-to-vitamin D₂ conversion. However, most of this evidence has been generated at laboratory scale, and translation to pilot or industrial production remains considerably less developed. Scale-up is not achieved simply by increasing the amount of material processed. The objective is to reproduce, as closely as possible, the process outcome obtained at a smaller scale, including yield, composition, and product quality, while increasing production rate. Relationships between laboratory and larger-scale extraction are also often specific to the raw material, target product, equipment, and operating conditions, which limits the applicability of universal scale-up criteria (62).

Laboratory optimization should therefore not be considered equivalent to process scalability. Conditions identified through response-surface methodology or other experimental optimization approaches describe performance within the investigated system and operating range, while scale-up also requires preservation of the physical conditions governing extraction. For solid-solvent processes, temperature, pressure, solvent velocity, and extraction-bed geometry are among the parameters that may need to remain controlled, whereas feed amount, solvent flow rate, and equipment dimensions increase with production scale (62). Heat and mass transfer and solvent distribution may consequently change with equipment size. In packed-bed systems, for example, non-uniform solvent contact or bed compaction can result in poorly extracted regions or preferential flow pathways (62).

A mushroom-specific example is provided by Morales et al. (63), who investigated the upscaling of SFE for the production of ergosterol-enriched *L. edodes* extracts. The larger-scale system used a 6-L extraction vessel and two 1.6-L separators. Extraction yield reached 0.53% after 3 h, while extending the process to 5 h resulted in only a small further increase, to 0.56%. The authors reported a lower extraction yield than that previously obtained with smaller SFE systems under similar conditions, but greater selectivity toward sterols, with ergosterol accounting for up to approximately 53% of the extract. The two separators also produced fractions with different sterol concentrations, with greater ergosterol enrichment in the first separator (63). Taken together, these results illustrate an important aspect of scale-up: changes in overall extraction yield do not necessarily parallel changes in target-compound enrichment, and both extraction and downstream fractionation need to be considered when evaluating process performance.

Scale-up requirements also depend on the extraction technology and the physical mechanism used to intensify mass transfer. DES-based UAE provides a useful mushroom-specific example. Patil et al. (64) first optimized extraction using 1 g of *A. bisporus* powder and subsequently increased the process to 5 g of mushroom powder in 100 mL DES before evaluating downstream purification. Although this represented a five-fold increase in processed material, the experiment remained at laboratory scale and therefore illustrates the distinction between laboratory process enlargement and pilot-scale validation.

This issue is particularly relevant to UAE because increasing treatment volume introduces additional constraints on acoustic energy delivery. In a review of scale-up studies involving natural products, Cauduro et al. (20) found that most UAE applications remained laboratory-based. Maintaining comparable power density and uniform energy dissipation becomes more difficult as processing volume increases, while transducer resistance at higher loads may further constrain operation. Larger-scale studies have consequently relied mainly on flow-through systems, including flow cells and probes coupled to recirculation, rather than direct enlargement of laboratory batch configurations (20). Translation is further complicated by incomplete process reporting, since ultrasonic power, frequency, amplitude, power density or intensity, reactor geometry, and temperature-control conditions are frequently omitted. These parameters are necessary to characterize the physical treatment delivered and therefore to compare laboratory and scaled UAE processes meaningfully.

Mushroom-specific scale-up data therefore remain limited to selected technologies and relatively modest changes in processing scale. Pilot-scale studies are still needed to establish whether recovery, selectivity, product composition, and productivity can be maintained under larger-scale operating conditions.

## 7 Techno-economic and environmental feasibility

Technical scalability alone does not establish environmental or economic sustainability. As emphasized by Kümmerer (65), “green” and sustainable are not equivalent concepts, and assessment should consider the complete process rather than individual operations or materials in isolation. This distinction is particularly relevant to mushroom valorization, where the use of a bio-residue, an alternative solvent, or an intensified extraction technology does not in itself demonstrate a lower overall environmental burden.

The most detailed mushroom-specific environmental assessment was reported by Moura et al. (66) for an ergosterol-enriched extract produced from mushroom bio-residues. Their cradle-to-gate LCA included lyophilization, grinding, UAE with ethanol, centrifugation, filtration, and solvent evaporation and recovery. The impacts were concentrated in a small number of operations. At the endpoint level, UAE accounted for approximately 31 to 32% of total potential damage, mainly because of ethanol use, while evaporation and lyophilization accounted for approximately 31 to 32% and 19 to 20%, respectively. Transport and grinding each contributed less than 1%. Equipment electricity consumption was the dominant input, contributing up to 70 to 85% of environmental impacts depending on the allocation approach (66).

The importance of electricity was confirmed when alternative scenarios were evaluated. Supplying the process with locally generated photovoltaic electricity reduced environmental impacts by up to approximately 50% relative to the base case, whereas osmotic dehydration before lyophilization produced a considerably smaller reduction of around 9%. Electricity requirements also had a stronger influence than ethanol consumption in most impact categories in the sensitivity analysis (66). These results shift attention from extraction yield alone to the complete sequence of operations required to obtain the final fraction. In the same process, ethanol recovery during evaporation generated an environmental credit by reducing the requirement for fresh solvent.

Economic assessment is less developed, although Pinto et al. (67) provide an early quantitative comparison of ergosterol- and vitamin D₂-enriched extracts obtained from *A. bisporus* bio-residues. Using Material Flow Cost Accounting together with environmental assessment, they estimated production costs of €2.43 g^−1^ for the ergosterol-enriched extract and €8.28 g^−1^ for the vitamin D₂-enriched extract. Recovery and reuse of ethanol reduced the respective costs by approximately 47 and 39%, with the estimated cost of the vitamin D₂-rich fraction decreasing to €5.04 g^−1^. These values refer to the specific laboratory-based process evaluated rather than established industrial production costs, but they show both the economic importance of solvent recovery and the additional cost associated with vitamin D₂ production. Importantly, the study did not determine whether this additional processing cost would be compensated by a higher market value of the vitamin D₂-rich product. Technical feasibility of photoconversion should therefore not be interpreted as evidence of economic viability.

Patil et al. (64) provide an example of the process considerations associated with alternative extraction solvents. A choline chloride:glycerol DES was successfully applied for UAE of vitamin D₂ from *A. bisporus*, and increasing the DES volume above 20 mL g^−1^ mushroom did not significantly improve extraction, indicating that higher solvent input was unnecessary under the conditions investigated. However, subsequent recovery and purification involved acetonitrile, water or hot-water treatment, *n*-hexane extraction, adsorbents or saponification, followed by solvent evaporation. Thus, while DES can provide an effective alternative medium for the extraction step, its potential environmental and process advantages need to be considered together with solvent requirements and the downstream operations required to obtain the target fraction.

Methodological choices can further influence environmental conclusions. In a systematic review of 36 LCA studies of natural-product extraction, Lian et al. (68) found that most assessments used cradle-to-gate or gate-to-gate boundaries, whereas only two adopted a cradle-to-grave perspective. The authors cautioned that omission of upstream inputs or downstream waste treatment can underestimate total impacts. They also highlighted the value of combining LCA with process simulation for industrial-scale assessment, while noting that predictions remain dependent on input data and assumptions concerning energy efficiency, solvent recovery, co-products, and process losses. Pilot-scale validation therefore remains important.

Further value may also be obtained from the solid material remaining after sterol extraction. Umaña et al. (53) applied UAE to *A. bisporus* stalks to recover ergosterol and phenolic compounds while evaluating the antioxidant activity of the resulting extracts. The extraction residues contained an average of 12.2 ± 1.7 g β-glucans/100 g dry matter, compared with 4.65 ± 0.30 g/100 g dry matter in the initial stalks. The authors associated this enrichment with the removal of other constituents during extraction and identified the residues as a potential source of biologically active polysaccharides. The study therefore shows that recovery of extractable bioactive compounds can simultaneously generate a *β*-glucan-rich residual fraction, providing a direct basis for considering ergosterol extraction within a broader sequential valorization strategy. Whether recovery and use of this additional fraction improve overall economic or environmental performance, however, remains to be established at process scale.

The available studies identify electricity demand, feedstock stabilization, solvent consumption and recovery, downstream purification, and the additional processing associated with vitamin D₂ production as important determinants of feasibility. Current environmental and economic assessments are still based predominantly on laboratory-scale processes, while industrially representative data remain scarce. Future evaluation should therefore connect pilot-scale mass and energy balances with product recovery, composition, downstream requirements, and value. Under these conditions, the most appropriate operating point may differ substantially from the conditions that provide maximum extraction yield in laboratory experiments.

## 8 Patented mushroom ergosterol and vitamin D₂ processing technologies

A targeted patent-landscape search identified 50 patents directly related to mushroom-derived ergosterol and vitamin D₂, together with eight related downstream-use applications. The latter covered food, nutritional or therapeutic applications of enriched mushroom materials, but did not primarily claim the enrichment process itself. The search was completed in August 2026 and covered UV photoconversion, irradiation equipment, whole mushrooms, powders, slurries, extracts, oils, product stabilization and approaches for increasing ergosterol availability.

The earliest patents on vitamin D₂ enrichment focused predominantly on *L. edodes*, especially dried *L. edodes* and *L. edodes* powder. Initial Japanese applications, dating in 1975 and 1978, described the UV irradiation of powdered or coarsely crushed *L. edodes*, followed by further grinding to obtain vitamin-D₂-rich ingredients (JPS51101160A; JPS5515766A). Subsequent inventions from 1981 and 1982 used thin slices of dried *L. edodes*, controlled UV wavelengths and conveyor-based systems to improve light exposure (JPS57189660A; JPS5921367A). Later designs introduced vibration, reflective chambers and lamps positioned around granular or powdered mushrooms, again mainly *L. edodes*, to obtain more uniform irradiation (1990, JP3171199B2, 1992, JPH0617496U). UV treatment was also integrated into artificial *L. edodes*-drying systems (1995, JP3619908B2), including equipment combining UV radiation with far-infrared heating (1997, JP3064247B2). Other applications from 1997 proposed irradiation devices for mushrooms before consumption and superheated-steam sterilization of vitamin-D₂- and ergosterol-rich *L. edodes* powder (JPH1146A; JPH1156288A). By 1998, the approach had expanded to living, growing or recently harvested mushrooms, especially *L. edodes* cultivated on logs, marking a transition from dried-material treatment toward controlled irradiation during cultivation and postharvest handling (JP2000157045A).

From the early 2000s, patent activity shifted from the treatment of dried *L. edodes* materials toward the controlled irradiation of fresh and whole mushrooms and the development of equipment suitable for commercial processing. Early applications used UV-B to enrich fresh or sliced *L. edodes* and other edible mushrooms under controlled temperature, humidity and exposure conditions (KR20030087209A, in 2002; KR100637833B1, in 2005), while conveyor systems were developed for continuous treatment of dried edible mushrooms (CN101057658A, in 2007). A major technological advance occurred in 2008 with the introduction of pulsed-light systems, which enabled rapid conversion of ergosterol into vitamin D₂ while limiting browning and other undesirable changes. These inventions *A. bisporus* and *L. edodes*, including atmospheric-plasma pretreatment before UV exposure (KR102172788B1, in 2018; KR102104597B1, in 2019). By 2021, equipment design had further progressed toward automated and uniform treatment, represented by systems using vibrating transparent surfaces and UV sources positioned above and below the mushroom material (KR102330075B1, in 2021).

While most early patents focused on increasing vitamin D₂ directly in whole mushrooms or mushroom powders, a smaller group of more recent inventions has combined photoconversion with extraction and separation. These approaches aim to obtain concentrated ergosterol or vitamin D₂ ingredients that can be more easily incorporated into foods, supplements or pharmaceutical products. Table 3 summarizes relevant patents published mainly within approximately the last decade.

**Table 3:** Selected patents registered from 2016 to 2026 using the keywords “ergosterol recovery,” “vitamin D₂ extraction” and “mushroom,” accessed in the EspaceNet Database and Google Patents in August 2026.

Year	Patent	Mushroom species	Description	Ref.
2016	KR101663143B1 / CN107108491B / US10751350B2 - Method for obtaining vitamin D2 from shiitake mushrooms	*L. edodes*	*L. edodes* powder is subjected to reflux extraction with 90–100% (v/v) ethanol. The resulting ergosterol-rich extract is then irradiated with UV light, at 254 nm, to convert ergosterol into vitamin D₂.	(75)
2018	CN108840811A - Vegetable-oil extraction of calciferol from mushroom powder	*A. bisporus*; *P. ostreatus*; *P. eryngii*; *H. erinaceus*; Shaggy ink cap (*Coprinus comatus*)	Dried mushrooms are ground, and the resulting powder is extracted with vegetable oil. The vitamin-D₂-enriched oil is separated from the mushroom solids by physical solid–liquid separation.	(76)
2019	CN109527121A/ AU2019400002B2 - Method for preparing mushroom vitamin D2 oil and use thereof	*A. bisporus*; *L. edodes*	Fresh mushroom slices are irradiated with combined UVB/UVC light to convert ergosterol into vitamin D₂ and are subsequently extracted with edible oil. The extraction vessel is vacuumized and filled with nitrogen to limit oxidation, isomerization and degradation, after which the mushroom solids are removed by solid–liquid separation.	(77)
2020	CN110156654A / CN110156654B – Preparation of a high-purity edible-mushroom vitamin-D₂ extract	*H. erinaceus* and maitake (*Grifola frondosa*)	An ergosterol-rich fraction is obtained from dried mushroom fruiting bodies by supercritical CO₂ extraction, followed by saponification, *n*-hexane extraction and molecular distillation. The purified fraction is then treated with intense pulsed light to convert ergosterol into vitamin D₂, yielding a high-purity vitamin D₂ extract.	(78)
2020	CN111642733A / KR102577063B1 / AU2021201901B2 – Increasing vitamin D₂ in mushrooms and preparing edible mushroom powder	*A. bisporus*; *L. edodes*; *P. ostreatus*	Vitamin D₂-enriched mushroom powder was produced by grinding and mechanically or enzymatically disrupting mushroom tissue, adjusting its moisture content, and irradiating the resulting particles with UV-B light (280–315 nm) while fluidized in a vibrating-bed reactor or boiling tower.	(79)
2021	CN112679402A - Low-Pollution Preparation Method for a *Sparassis crispa* Vitamin D₂ Extract*	*S. crispa*	Ground fruiting bodies are ultrasonically dispersed in an alkaline ethanolic solution and simultaneously treated with a pulsed electric field and combined UVB/UVC irradiation. Subsequent saponification and electrochemical removal of heavy metals yield a vitamin-D₂-rich extract with reduced heavy-metal contamination.	(80)
2023	CN112624947B - Method for Extracting High-Content Vitamin D₂ from *Sparassis crispa* Fruiting Bodies	*S. crispa*	Ground and sieved fruiting bodies are ultrasonically dispersed in water containing a water-soluble fluorescent nanomaterial and treated with a pulsed electric field under light irradiation. Following alkaline saponification, vitamin D₂ is extracted with absolute ethanol.	(81)
2023	CN117209551A - Method for extracting ergosterol from *Lentinus edodes* stems by using natural eutectic solvent**	*L. edodes*	Ultrasound-assisted method for recovering ergosterol from shiitake mushroom stems using NADESs. The solvent consists of L-proline and levulinic acid at a 1:2 molar ratio with 5% water. Freeze-dried, powdered mushroom stems are mixed with the solvent at a solid-to-liquid ratio of 1:40 g/mL and extracted at 50 °C and 200 W for 80–95 min.	(82)
2026	CN121717747A - Method for converting ergosterol into vitamin D₂ using DES and method for enriching vitamin D₂**	*A. bisporus*	Material is mixed with the NADES and subjected to ultrasonication. NADES is composed of menthol/octanoic acid, menthol/decanoic acid or octanoic acid/decanoic acid. Extraction conditions are 40–60 °C, 30–50 kHz and 40–80 min. The extract is directly irradiated with UV-B, followed by pH-triggered solvent switching and vitamin-D₂ recovery.	(83)

*The application was withdrawn, although its technical disclosure remains relevant. **Status: pending.

These patents demonstrate a gradual transition from simple solvent extraction toward more integrated processes combining extraction, ergosterol enrichment, photoconversion and product purification. The earliest ones used ethanol reflux extraction of *L. edodes* powder before UV conversion of the extracted ergosterol. The patents filed in 2018 introduced three different strategies: direct extraction of vitamin D₂ into food-grade vegetable oils, combined UVB/UVC treatment followed by edible-oil extraction, and production of high-purity extracts using supercritical CO₂, saponification, solvent extraction and molecular distillation. More recent inventions involving cauliflower mushroom (*Sparassis crispa*) incorporated ultrasound, pulsed electric fields and UV irradiation to improve cell disruption and ergosterol conversion. One of these applications additionally included electrochemical removal of heavy metals. The edible-oil processes appear suitable for direct food application, whereas the methods involving saponification, organic solvents, molecular distillation or nanomaterials are more complex and may require additional purification, safety evaluation and economic assessment before industrial implementation.

## 9 Conclusions and future directions

Mushrooms and their processing side streams provide versatile feedstocks for the recovery of ergosterol and the production of vitamin D₂-rich fractions. The literature reviewed here shows that no single extraction technology is superior across all relevant criteria. UAE and MAE can substantially shorten extraction, PLE and SFE provide different balances between recovery and selectivity, and DES/NADES systems introduce opportunities for solvent tailoring, reuse and integration with subsequent photoconversion. The appropriate technology therefore depends on the intended product rather than on maximum extraction yield alone. Across these approaches, matrix preparation, extraction conditions and downstream processing remain closely interconnected.

A particularly important development is the shift from isolated extraction toward integrated ergosterol recovery and vitamin D₂ production. SFE-based sequential processing and, more recently, NADES systems capable of supporting both extraction and photoconversion demonstrate this progression. However, integration introduces additional requirements that have received considerably less attention than extraction itself. Photoconversion must be evaluated in terms of photon delivery, irradiation geometry, conversion selectivity and product stability, while extraction media must also be assessed for their compatibility with recovery, reuse and downstream formulation. Future optimization should therefore consider recovery, enrichment, productivity and resource demand simultaneously rather than maximizing a single response.

Scale remains the principal barrier between the laboratory evidence and practical production. Mushroom-specific studies beyond laboratory scale are scarce, and available examples show that extraction yield and selectivity do not necessarily change in parallel when processing conditions or equipment scale are affected. The patent landscape nevertheless indicates sustained technological interest and a progression from simple UV treatment toward more controlled irradiation systems and integrated extraction, conversion and purification strategies. This contrast is significant: technological concepts for commercial processing are already being developed, but publicly available experimental evidence on pilot-scale performance, energy and solvent balances, product consistency and process economics remains limited. The mushroom-specific techno-economic and life-cycle assessment conclusions currently rely on only a small number of laboratory-based assessments and should not be generalized to industrial production without validation.

Future development should therefore focus less on demonstrating additional laboratory extraction concepts and more on validating complete processing routes. Pilot-scale studies should connect feedstock preparation, selective recovery, photoconversion, solvent management and downstream fractionation with techno-economic and life-cycle assessment.

Sequential valorization of the remaining mushroom biomass, including β-glucan-rich residues, may further improve resource utilization, but its benefit must be demonstrated rather than assumed. Progress toward standardized process reporting and integrated technical, economic and environmental evaluation will ultimately determine whether the high recoveries and conversion efficiencies achieved experimentally can be translated into viable production of mushroom-derived ergosterol- and vitamin D₂-rich ingredients. Minimum quantitative reporting should include processed mass and throughput, target recovery and concentration, solvent-to-feed ratio and solvent-recovery percentage, specific energy demand, extraction productivity, and mass balances across purification steps. UV studies should report wavelength and spectral range, irradiance, exposure time and fluence/dose, source-to-sample distance, exposed area or film thickness, mixing, temperature and moisture, initial ergosterol content, vitamin D₂ yield/conversion, and lumisterol₂/tachysterol₂ or other relevant photoproducts. Product-stability studies should quantify vitamin D₂ retention under defined temperature, light, oxygen, packaging and storage-time conditions rather than relying only on concentrations measured immediately after irradiation. Sequential valorization of the remaining mushroom biomass, including β-glucan-rich residues, should also be incorporated into process mass balances and tested for its contribution to revenue and environmental allocation. The 12.2 ± 1.7 g β-glucans 100 g^−1^ dry matter reported in post-UAE *A. bisporus* stalk residues (53), compared with 4.65 ± 0.30 g 100 g^−1^ in the initial stalks, illustrates the potential value of this co-product, but its process-scale benefit remains to be demonstrated. Therefore, future work should use these standardized quantitative metrics to establish technology- and product-specific benchmarks.
